# Cross-cultural adaptation of the Social and Emotional Questionnaire on Dementia for the Brazilian population: Adaptação transcultural do Questionário Social e Emocional na Demência para a população brasileira

**DOI:** 10.1590/1516-3180.2014.00180501

**Published:** 2015-08-03

**Authors:** Tatiana Belfort, Jessica Bramham, José Pedro Simões, Maria Fernanda Barroso de Sousa, Raquel Luiza dos Santos, Marcela Moreira Lima Nogueira, Bianca Torres, Rachel Dias Lopes da Rosa, Marcia Cristina Nascimento Dourado

**Affiliations:** I BSc. Master’s Student, Center for Alzheimer’s Disease and Related Disorders, Institute of Psychiatry, Universidade Federal do Rio de Janeiro (UFRJ), Rio de Janeiro, Brazil.; II PhD. Senior Lecturer, Clinical Neuropsychology, School of Psychology, University College Dublin, Dublin, Ireland.; III PhD. Professor, Department of Sociology and Political Science, Universidade Federal de Santa Catarina (UFSC), Florianópolis, Brazil.; IV MSc. Doctoral Student, Center for Alzheimer’s Disease and Related Disorders, Institute of Psychiatry, Universidade Federal do Rio de Janeiro (UFRJ), Rio de Janeiro, Brazil.; V BSc. Psychologist and Specialist in Aging Studies, Center for Alzheimer’s Disease and Related Disorders, Institute of Psychiatry, Universidade Federal do Rio de Janeiro (UFRJ), Rio de Janeiro, Brazil.; VI PhD. Collaborating Professor, Center for Alzheimer’s Disease and Related Disorders, Institute of Psychiatry, Universidade Federal do Rio de Janeiro (UFRJ), Rio de Janeiro, Brazil.

**Keywords:** Awareness, Cross-cultural comparison, Questionnaires, Dementia, Emotions, Conscientização, Comparação transcultural, Questionários, Demência, Emoções

## Abstract

**CONTEXT AND OBJECTIVE:**

Impairments in social and emotional functioning may affect the communication skills and interpersonal relationships of people with dementia and their caregivers. This study had the aim of presenting the steps involved in the cross-cultural adaptation of the Social and Emotional Questionnaire (SEQ) for the Brazilian population.

**DESIGN AND SETTING:**

Cross-cultural adaptation study, conducted at the Center for Alzheimer’s Disease and Related Disorders in a public university.

**METHODS:**

The process adopted in this study required six consecutive steps: initial translation, translation synthesis, back translation, committee of judges, pretesting of final version and submission to the original author.

**RESULTS:**

In general, the items had semantic, idiomatic, conceptual and experiential equivalence. During the first pretest, people with dementia and their caregivers had difficulties in understanding some items relating to social skills, which were interpreted ambiguously. New changes were made to allow better adjustment to the target population and, following this, a new pretest was performed. This pre-test showed that the changes were relevant and gave rise to the final version of the instrument. There was no correlation between education level and performance in the questionnaire, among people with dementia (P = 0.951).

**CONCLUSION:**

The Brazilian Portuguese version of the Social and Emotional Questionnaire was well understood and, despite the cultural and linguistic differences, the constructs of the original version were maintained.

## RESUMO

**CONTEXTO E OBJETIVO:**

O comprometimento do funcionamento social e emocional pode afetar as habilidades de comunicação e relações interpessoais das pessoas com demência e de seus cuidadores. Este estudo tem como objetivo apresentar as etapas envolvidas na adaptação transcultural do Questionário Social e Emocional (SEQ) para a população brasileira.

**TIPO DE ESTUDO E LOCAL:**

Estudo de adaptação transcultural, realizado no Centro de Doença de Alzheimer e Transtornos Relacionados de uma universidade pública.

**MÉTODOS:**

O processo adotado neste estudo exige seis etapas consecutivas: tradução inicial, síntese de tradução, “retrotradução”, comitê de julgadores, pré-teste da versão final e a submissão ao autor original.

**RESULTADOS:**

Em geral, os itens apresentaram equivalência semântica, idiomática, conceitual e vivencial. Durante o primeiro pré-teste, pessoas com demência e seus cuidadores apresentaram dificuldades de compreensão de expressões nos itens referentes à sociabilidade, que foram interpretadas com duplo sentido. Novas alterações foram feitas para maior adequação à população-alvo, sendo em seguida realizado um novo pré-teste, que demonstrou a pertinência das adequações, originando então a versão final do instrumento. Não foi encontrada correlação entre o desempenho no questionário pelas pessoas com demência e a escolaridade (P = 0.951).

**CONCLUSÃO:**

A versão em português brasileiro do Social Emotional Questionnaire foi bem compreendida e, apesar das diferenças culturais e linguísticas, os constructos da versão original foram mantidos.

## INTRODUCTION

Awareness of deficits has been defined as the capacity to recognize changes caused by impairments relating to the disease process.^1^^,^^2^ It may be expressed at different levels, including the ability to monitor immediate performance, make evaluative judgments about functioning in a given domain and reflect on the nature and impact of a diagnosis or health condition.^3^ Unawareness of deficits has been commonly reported as a clinical feature of neurodegenerative disorders, but there are conflicting results regarding the frequency and its relationship with disease severity.^4^^,^^5^ Impaired awareness of deficits may be present in the early stages, ranging from very mild to very severe.^6^

Awareness of social and emotional functioning relates to essential aspects of social interactions, such as the capacity to understand other people and oneself, and the capacity to comprehend interpersonal relationships.^7^ Under different pathological conditions, the level of impaired awareness of social and emotional functioning is related to the various degenerative patterns.^8^ In Alzheimer’s disease, there are common alterations, such as apathy, disinhibition and reduced mental flexibility. However, recognition of emotions may be preserved in Alzheimer’s disease, in comparison with vascular dementia.^8^^,^^9^ Under frontotemporal dementia, individuals might present behavior that is incompatible with rules and social norms, along with difficulty in understanding emotions.^10^

Impairment of social and emotional functioning may damage communication skills and interpersonal relationships, thereby directly influencing the activities of daily living and the quality of life of people with dementia and their caregivers.^11^ The instruments for assessing dementia mainly focus on the cognitive and functional domains.^12^ There is a lack of availability of appropriate specific instruments for measuring social and emotional functioning.^7^ Therefore, a cross-cultural adaptation process needs to be undertaken in order to improve the knowledge about recognition of social and emotional functioning among Brazilian people with dementia.^13^

The Social and Emotional Questionnaire (SEQ) was originally developed in English by Bramham et al.^11^ to evaluate social and emotional functioning. The questionnaire was used to assess behavioral reports relating to specific acquired social difficulties following prefrontal cortex damage in adults, and it particularly focused on recognition of emotion and empathic reactions. It has been validated for other populations such as older adults with dementia, young adolescents and people with anorexia nervosa.^9^^,^^14^^,^^15^

## OBJECTIVE

This study had the aim of presenting the cross-cultural adaptation process of the Social and Emotional Questionnaire (SEQ) for people with dementia in the Brazilian population.

## METHODS

The SEQ comprises 30 items distributed into five factors: emotion recognition, empathy, social conformity, antisocial behavior and sociability.^11^ The emotion recognition subscale includes items that assess the perceived ability to recognize basic emotions in others (happiness, anger, sadness, fear and disgust).^11^

The ratings are scored on a five-point Likert scale, from “strongly disagree” (1) to “strongly agree” (5). The “people with dementia” and “caregivers” versions are essentially identical.^11^^,^^14^ People with dementia rate their socioemotional functioning with regard to their ability to recognize emotions, the extent of their empathetic reactions and their behavior in social situations. Caregivers also complete the SEQ, in relation to the current functioning of people with dementia.^7^ The score is based on the degree of discrepancy between results from people with dementia and from their caregivers.^7^^,^^11^

The first description of psychometric properties was made in relation to brain injury patients.^11^ The items that loaded most highly in each factor were used to form five subscales: emotion recognition (5, 8, 12, 18, 23); empathy (3, 9, 15, 20, 30); social conformity (11, 14, 25); antisocial behavior (1, 6, 13, 24); and sociability (4, 16, 17, 19, 21, 27, 29). The total score for the patient version of the SEQ had good correlation with the patients’ total score on the Patient Competency Rating Scale (PCRS) (P = 0.01) and the total score for the proxy version had strong correlation with the proxy-version total score of PCRS (P < 0.01). Based on these results, the SEQ was considered to be a valid instrument for assessing the recognition of social and emotional functioning among brain injury patients.^11^

Nelis et al.^7^ conducted the first validation study on the SEQ among people with dementia. Varimax rotation yielded three interpretable solutions: the 11-item emotional recognition and empathy (ERE) domain (3, 5, 8, 9, 11, 12, 15, 18, 20, 23, 30), the 7-item social relationship (SR) domain (16, 17, 19, 25, 26, 28, 29) and the 6-item prosocial behavior (PB) domain (2, 4, 6, 10, 13, 22). There were small but significant differences between self-ratings and caregivers’ ratings for all the domains: ERE: F(1, 96) = 17.98, P < 0.001; SR: F(1, 96) = 4.65, P < 0.03; and PB: F(1, 96) = 4.10, P < 0.04. According to these data, people with mild dementia have reduced awareness of social functioning, especially with regard to emotional recognition and empathy.^7^

### Cross-cultural adaptation

The cross-cultural adaptation process was based on the protocol proposed by Beaton et al.,^16^ after obtaining permission from the original author of the scale. The following consecutive steps were implemented: initial translation, translation synthesis, back translation, committee of judges, pretesting of the final version and submission to the author of the original instrument.^16^ The study was approved by the Research Ethics Committee of the Institute of Psychiatry (IPUB) of the Universidade Federal do Rio de Janeiro (UFRJ). All the participants with dementia and their caregivers signed the informed consent statement.

### Initial translation

The initial translation of the original English version of the instrument into Brazilian Portuguese was performed by three independent translators who were fluent in both English and Portuguese (T1, T2 and T3). Translators T1 and T2 were professionals who did not have any knowledge of the content, in order to try to reduce the possible influence of academic words.^16^ The third translator (T3) was a psychologist with experience in the field, who was capable of identifying the constructs of the items of the instrument.^16^^,^^17^ This step maintained operational equivalence, thus keeping the characteristics of using the SEQ, such as the vehicle and formatting of questions and instructions, along with the way of applying the instrument.^16^^,^^18^

### Translation synthesis

For the second stage, the three translations were analyzed by two psychologists (doctoral students) who had not participated in the previous step, but had mastered the constructs of the instrument.^13^ The synthesis was based on the results from the translations.^16^

### First back translation

The third stage was back translation, which formed a quality control on the translation that been produced. The back translation was performed on the translated synthesis by a bilingual psychologist, who was a doctoral student in the field of deficit awareness, but had no knowledge of the original scale.^16^^,^^19^ The objective was to check for any type of inconsistency or conceptual error in the translations.^16^

### Committee of judges

For the fourth stage, a committee of judges was organized to evaluate the equivalence between the original version and the Brazilian version of the instrument.^16^ The committee was composed of eight healthcare professionals: one neurologist, two psychiatrists, three physiotherapists and two psychologists. The committee evaluated the semantic, idiomatic, conceptual and experiential equivalence.^16^ These different forms of equivalence related to the following: the essence of the content with regard to literal translation of the words (semantic equivalence); colloquialisms and linguistic expressions (idiomatic equivalence); the concept of the phenomenon assessed (conceptual equivalence); and the culture experienced (experiential equivalence).^20^ After the committee had expressed its views, a new synthesis in Portuguese was put forward, in which the changes suggested by the judges were added.^16^^,^^21^

### Pretesting of the final version

For the fifth stage, a pretest with the new translated synthesis was applied in order to ascertain the degree of comprehension of the instrument among the target population.^16^^,^^21^ The sample comprised 30 participants, i.e. 15 people with dementia and their caregivers. The participants had been diagnosed with possible or probable Alzheimer’s disease in accordance with the Diagnostic and Statistical Manual of Mental Disorders, Fourth Edition (DSM-IV-TR).^22^ Only individuals with mild and moderate Alzheimer’s disease, corresponding to Clinical Dementia Rating (CDR) and Mini-Mental State Examination (MMSE) scores of 12-26 were included in the study.^23^^,^^24^

The pretesting was performed at the Center for Alzheimer’s Disease and Related Disorders (CDA) of IPUB/UFRJ, between September 2013 and March 2014. The assessment method was different for caregivers and people with dementia. This distinction was made in an attempt to minimize the impact of the cognitive deficits among people with mild and moderate dementia with regard to comprehending the items. The caregivers provided responses to the SEQ in a self-applied manner, while the people with dementia were assessed through face-to-face interviews. The people with dementia and their caregivers answered the questionnaire on the same day, which took approximately seven minutes to do.

Some comprehension difficulties were observed from the pretesting process. The final version was then resubmitted to a new committee of judges, composed of five specialist psychologists who were fluent speakers of Portuguese and English, with the objective of achieving better equivalence between the original items and the adapted version.^16^ After further adaptations, the final version was built and a new pretest was conducted. The new sample was composed of 20 participants, i.e. 10 people with dementia and their caregivers, with the same inclusion and exclusion criteria as in the first sample. All the participants were able to understand and answer the final version.

### Second back translation

The second back translation was performed by a different professional, who had full knowledge of the subject and full skills in Portuguese and English.

### Submission to the author of the original instrument

After the pretesting adjustments had been made, the final Brazilian Portuguese version was sent to the author of the original instrument.^16^ Particularly with regard to evaluating the second back translation, the participation by the author of the original instrument helped to ensure that the adapted version was compatible with the original.^16^^,^^19^

### Statistical analysis

Simple statistics were included to analyze the effect of education on comprehension of the SEQ items. The statistical analyses were performed using the SPSS software for Windows, release 21.0. We used Spearman’s correlation to investigate the relationships between education level and dementia in both pretests of the translated SEQ.

## RESULTS

The sociodemographic characteristics of the samples are presented in Table 1.

**Table 1.**
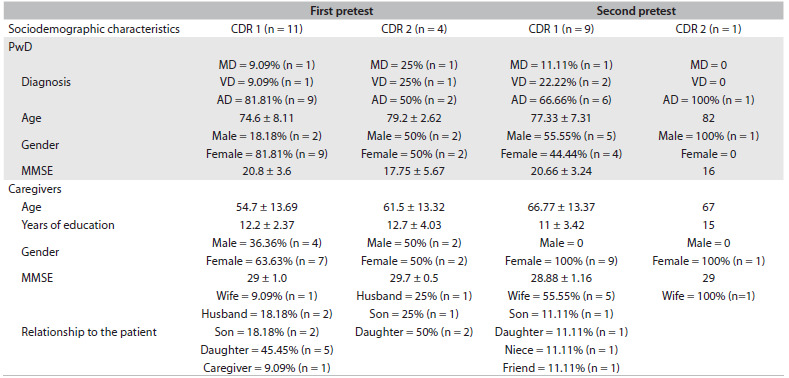
Sociodemographic characteristics of pretest samples

CDR = Clinical Dementia Rating; 1 = Mild impairment; 2 = Moderate impairment; PwD = People with Dementia; MD = Mixed dementia; VD = Vascular dementia; AD = Alzheimer’s dementia; MMSE = Mini Mental State Examination.

The original questions of the SEQ and the results from the initial translation, translation synthesis, back translation and submission to the author of the original instrument are presented in Table 2. The results from the evaluation by committee of judges and from the pretesting of the final version are described within the text, below.

**Table 2.**
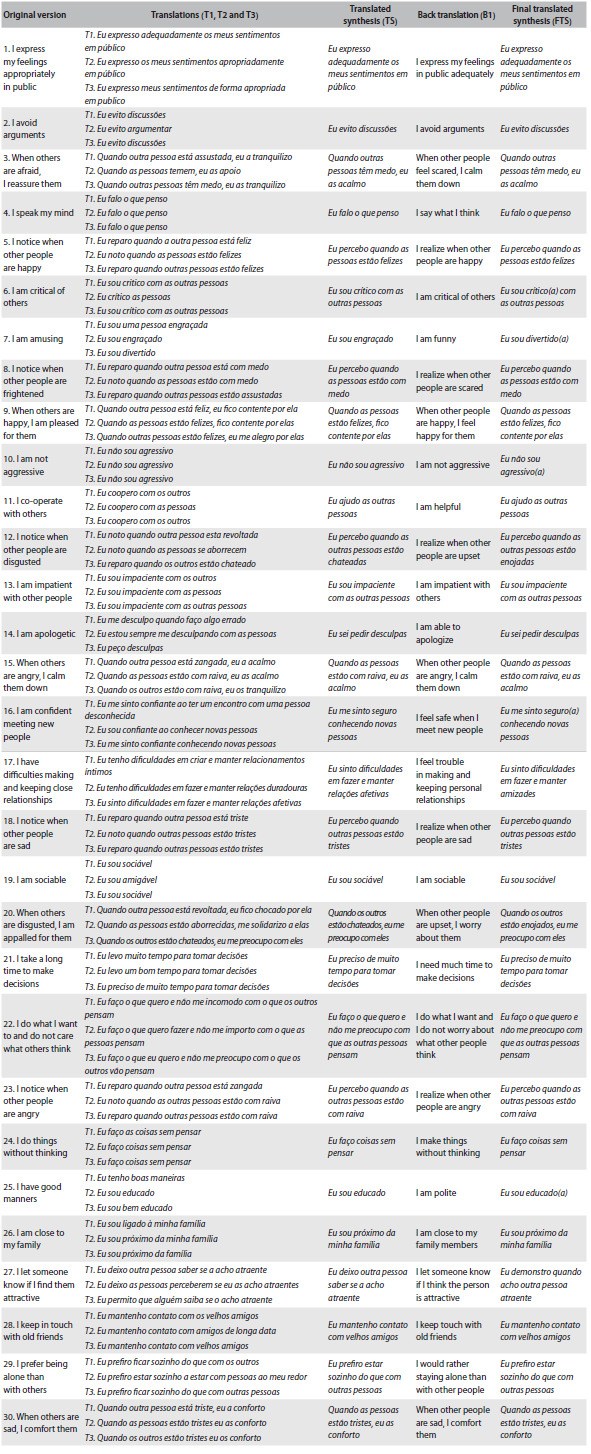
Steps of the cross-cultural adaptation process

T1, T2 and T3 = Translation 1, Translation 2 and Translation 3; TS = Translated synthesis; B1 = Back translation; FTS = Final translated synthesis.

During the development of the first translated synthesis, we tried to achieve a consensus with regard to identifying the best translation. The psychologists responsible for the translation synthesis noticed that the translation performed by T1 was more formal than the others, which would possibly affect the comprehension of the instrument. Therefore, T2 and T3 were prioritized because the language that they used was more similar to the words used in Brazilian daily life. The back translation had good equivalence regarding the reference framework and general meaning, in comparison with the original instrument.

The committee of judges analyzed all the stages: the translations, the translation synthesis and the back translation. The items had semantic, idiomatic, conceptual and experiential equivalence. Operational adjustments were proposed in an attempt to aid in applying and correcting the instrument. In items 6, 7, 10, 16 and 25, we included the female gender in brackets to help in identifying the participant in each sentence. We added an area for counting the results from each individual column, at end of the questionnaire, in the versions applied to both the people with dementia and their caregivers. Furthermore, we decided to include an area for the total score (sum of the columns). In order to improve the comprehension of the total scores, we also added an area for the scores relating to the people with dementia and their caregivers, and for the formula for calculating the discrepancy between people with dementia and their caregivers, along with the cutoff points.

In the pretests, the participants were divided according to the severity of their disease, as assessed using the Clinical Dementia Rating.^23^ During the first pretest, the people with dementia and their caregivers had difficulty in understanding items 7, 17 and 27. In item 7, “*Eu sou engraçado*”, the word “*engraçado*” was replaced by the synonym “*divertido*”, since the first word was understood by people with dementia in a pejorative way, i.e. someone who makes jokes inappropriately. In item 17: “*Eu sinto dificuldades em fazer e manter relações afetivas*”, 80% of the people with dementia (81% CDR1; 75% CDR2) and 46.6% of the caregivers (54.5% CDR1; 25% CDR2) interpreted this sentence as relating to sexual intercourse. Since this is an item of sociability, the expression “affective relationships” was replaced by “*fazer e manter amizades*”. In item 27, “*Eu deixo outra pessoa saber se a acho atraente*”, 46.6% of the people with dementia (45.5% CDR1; 50% CDR2) and 33.33% of the caregivers (36.3% CDR1; 25% CDR2) had difficulty in understanding the meaning of the sentence. Therefore, the sentence was modified to “*Eu demonstro quando acho outra pessoa atraente ou interessante*”. After analysis by a new committee of judges, changes were made in order to adapt the inappropriate words to the target population. A new version of the instrument was built and a second pretest was performed. In this new version of the SEQ, all the items were correctly understood both by the people with dementia and by their caregivers.

There was no correlation between education level and dementia, either in the first pretest (*r* = - 0.343, P = 0.210) or in the second pretest (*r* = 0.023, P = 0.951) of the translated SEQ.

The Brazilian version was sent to the author of the original instrument, who made some observations about the translations of items 1, 12 and 20. In item 1, the word “*appropriately*” was translated as “*adequadamente*” (adequately). The author considered that there was a slight difference between the two words, but she believed that the sense was still maintained. In items 12 and 20, the word “*disgusted*” had been translated as “*chateado*” (upset), but the author explained that those items relate to emotional recognition and are based on the five basic emotions (happiness, sadness, anger, fear and disgust). Therefore, the word “*disgusted*” was changed to “*enojado*”. Although this is not a common word in the Brazilian context, we decided to keep the main characteristics of the instrument. The modifications suggested by the author of the original instrument were made and the final version of SEQ was thus concluded (Table 2).

## DISCUSSION

Unawareness of social and emotional functioning among people with dementia may have implications such as understanding other people and themselves, comprehension of interpersonal relationships and the ability to respond empathically to the emotions of other people.^7^^,^^15^ More broadly, unawareness of social and emotional functioning is associated with poor treatment outcome and high burdens on caregivers.^15^ The SEQ is an instrument that has been shown to be sensitive to social and emotional unawareness in relation to some diseases, including dementia.^15^

The objective of this study was to culturally adapt the SEQ for the Brazilian population, while maintaining the original concept of the instrument and taking the distinct aspects of cultural and language into consideration.^19^ Cross-cultural adaptation of a research instrument is an important step in scientific investigations. Errors at this stage may misrepresent the original intention of the instrument, thereby compromising the validity and reliability of the study.^17^ The language differences and, especially, the cultural specificities of each country show why adaptation of an instrument is much more complex than merely translating it. This process needs to be a combination of literal translation of the words and sentences with rigorous consideration of the cultural context and lifestyle of the target population.^18^

The cross-cultural adaptation of the SEQ to Brazilian Portuguese had semantic, idiomatic, conceptual and experiential equivalence and it seems that the translated words and expressions kept their original characteristics.^19^ During the pretests, we observed that people with dementia had difficulty in understanding some items relating to social skills and emotional recognition and empathy. These items were interpreted ambiguously and a new committee of judges was necessary in order to discuss the equivalence of words. The statistical analysis showed that there was no correlation between education level and dementia in the translated questionnaire. Thus, we can suggest that the difficulties with some items may have been due to cognitive impairment or the presence of unawareness of any deficit caused by the disease. Nelis et al.^7^ indicated that people with early-stage dementia show reduced awareness of their social functioning, particularly with regard to emotion recognition and empathy, but also in their social relationship skills and prosocial behavior.^7^

This study had some limitations. The sample selection did not highlight any proportional distribution of the different levels of severity of the disease, which hindered the comparison between groups. If the distribution had been more homogeneous, better distinction of the capacity to comprehend the instrument among the people with mild and moderate dementia would have been possible. We also only ran simple statistical tests, given that the objective of this study was to present the first steps of the process of validation of the instrument (translation, adaptation and semantic equivalence) for the Brazilian population.^25^ The subsequent steps, aimed towards investigating the psychometric properties, are being processed and will be presented in future studies.^26^

## CONCLUSION

The cross-cultural adaptation process of the Social and Emotional Questionnaire demonstrated that this instrument was well understood by the Brazilian population. Regardless of the cultural and linguistic differences, the main constructs of the original version were maintained.
